# The Influence of Different Stresses on Glomalin Levels in an Arbuscular Mycorrhizal Fungus—Salinity Increases Glomalin Content

**DOI:** 10.1371/journal.pone.0028426

**Published:** 2011-12-12

**Authors:** Edith C. Hammer, Matthias C. Rillig

**Affiliations:** 1 Microbial Ecology, Department of Biology, Lund University, Lund, Sweden; 2 Institut für Biologie, Freie Universität Berlin, Berlin, Germany; University of Minnesota, United States of America

## Abstract

Glomalin is a glycoprotein produced by arbuscular mycorrhizal (AM) fungi, and the soil fraction containing glomalin is correlated with soil aggregation. Thus, factors potentially influencing glomalin production could be of relevance for this ecosystem process and for understanding AM fungal physiology. Previous work indicated that glomalin production in AM fungi may be a stress response, or related to suboptimal mycelium growth. We show here that environmental stress can enhance glomalin production in the mycelium of the AM fungus *Glomus intraradices*. We applied NaCl and glycerol in different intensities to the medium in which the fungus was grown *in vitro*, causing salinity stress and osmotic stress, respectively. As a third stress type, we simulated grazing on the extraradical hyphae of the fungus by mechanically injuring the mycelium by clipping. NaCl caused a strong increase, while the clipping treatment led to a marginally significant increase in glomalin production. Even though salinity stress includes osmotic stress, we found substantially different responses in glomalin production due to the NaCl and the glycerol treatment, as glycerol addition did not cause any response. Thus, our results indicate that glomalin is involved in inducible stress responses in AM fungi for salinity, and possibly grazing stress.

## Introduction

Glomalin is a glycoprotein produced by arbuscular mycorrhizal fungi (AMF). The soil organic matter fraction called glomalin-related soil protein (GRSP; [1] is hypothesized to at least partly consist of glomalin, and GRSP has been shown to be well correlated with soil aggregation [2]. Glomalin has been characterized as a putative homolog of heat shock protein (hsp) 60 [3], but little is known about the ecophysiology of glomalin production so far, and it is not clear why AMF produce this compound.

Driver and Rillig [4] suggested that a major proportion, more than 75% of the glomalin produced in sterile *in vitro* cultures, were mycelium-bound and not released into the environment, in this case the growth medium. The location of the MAb32B11-immunoreactive protein was confirmed to be predominantly hyphal- and spore wall bound instead of being found in the cytoplasm [5]. Glomalin levels in soil and *in vitro* cultures were negatively correlated with hyphal length [6], [7], suggesting that its production might be a stress response. A common stress in soils is grazing by soil biota, and AMF were found to have lower palatability than other fungi to soil mesofauna [8], and if the only food source, reduced collembolan fecundity significantly [9]. This led to the hypothesis that glomalin might be involved in grazing stress avoidance or grazing defence [10].

To address the question if glomalin production is an inducible stress reaction to grazing or other general stresses, we applied three different stress factors to the AM fungus *G. intraradices* in *in vitro* cultures: (a) grazing stress represented by mechanical damage with scissors; (b) water stress by lowering the water potential of the growth environment by glycerol addition to the medium; (c) salinity stress by addition of NaCl. These three stresses are common and relevant in soils as soils generally contain hyphal consumers; water stress occurs in most soils regularly in periods between rain events, and elevated salinity is caused even in non-saline soils as the soil solution becomes more concentrated during drying. All stress factors were applied as gradients to examine not only the existence of responses, but also their shape.

We hypothesized that an increase in stress factors would lead to increased glomalin production in a sterile *in vitro* culture.

## Materials and Methods

### Experimental design

We used two-compartment split-plates of root organ cultures of *Daucus carota* L. inoculated with *Glomus intraradices* Schenk & Smith (DAOM 197198) [11] - recently recommended to be renamed to *G. irregulare* [12] - as described in [13]. The minimal (M) medium used for cultivation contains amongst other mineral nutrients 0.9 µg g- P and 0.5 µg g- Na, as well as 3 g L- phytagel and 10 g L- sucrose in the root compartment [14]. The M medium in the hyphal compartment (lacking phytagel and sucrose) has an ionic strength of 13 mOsm. By using *in vitro* systems of *G. intraradices*, we could avoid cross-reaction of the antibody with proteins produced by other soil microorganisms or humic acids that could interfere with glomalin measurements [15], [16]. Since these potentially interfering entities are absent, we refer to the protein detected by the monoclonal antibody (see below) as glomalin.

The same numbers of plates with all treatments were prepared from each mother culture, and randomly chosen for each treatment and treatment level. The hyphal compartment (HC) was filled 30 days after plate establishment with liquid M medium lacking both sucrose and phytagel, and was amended with the following three treatment series. The clipping treatment was applied on the edges of the hyphal front two months after application of the liquid medium. Three sequential clipping events, each between 4 and 20 cuts, were applied at one week intervals, resulting in 12 to 60 cutting injuries of the mycelium (n = 4). Four control plates to the clipping treatment were opened and handled in the same way, but not clipped. This handling did not affect glomalin production or hyphal growth. For the water stress treatment, glycerol was added to the HC at the start of the experiment in concentrations ranging between 50 and 750 mM (n = 4). NaCl, which has an osmolarity twice that of glycerol because it dissolves into two ions, was added in concentrations between 10 and 500 mM (n = 4). Those were the ranges of osmolarity that *G. intraradices* tolerated and still sustained growth in a preliminary study. Each treatment series had four control plates, which did not differ from each other.

Plates were harvested after 3.5 months of growth in the hyphal compartment. Spore to hyphae ratio was recorded by visually categorizing amounts of spores and hyphae into 5 categories. Hyphae were freeze-dried and mycelium dry weight was recorded. The solid medium of the root compartment was solubilised in Na citrate (10 mM) [17] and root dry weight was recorded.

### Glomalin assays

Hyphal samples were macerated with 500 µl glass beads in 2 ml microfuge tubes containing 50 mM Na citrate extraction buffer at pH 8. For protein extraction, samples were autoclaved at 121°C in each 1.5 mL 50 mM Na citrate for 60 min in three cycles, and supernatants were collected. Determination of the glomalin concentration in the sample extracts with indirect ELISA was performed using the monoclonal antibody MAb32B11 as described in [2]. Each 50 µl sample extract diluted in phosphate buffered saline (PBS, for pH stabilization; 1∶1) was dried overnight at 35°C on U-shaped wells of polyvinyl-chloride Dynex micro-plates. Remaining binding sites were blocked by 2% non-fat milk in PBS for 15 min. Samples were incubated with the monoclonal glomalin antibody MAb32B11 in PBS (1∶30), which was originally produced against spores of *G. intraradices* [2], for one hour. After incubation, wells were washed three times with PBS-Tween 20 (0.02%). The glomalin- MAb32B11 complex was incubated with biotinylated antimouse IgM antibody in 1% BSA (1∶1000) for one hour, followed by three washes with PBS-Tween 20. The complex was incubated with extravidin alkaline phosphatase (Sigma E2636) in 1% bovine serum albumin (BSA; 1∶1000) for one hour, washed three times with PBS-Tween 20 and once with Tris buffered saline-Tween 20. The wells were incubated with the color developer diethanolamine buffer in 0.01%MgCl_2_ aqueous solution, pH 9.8, for exactly 30 minutes. Incubation steps were performed on a rotary shaker. 30 minutes after application of the color developer, color intensity was read at 405 nm wavelength with a microplate spectrophometer (Benchmark Plus, Bio-Rad, Germany). A standard curve for ELISA was prepared in the range of 5–40 ng glomalin protein isolated from a standard soil in Montana, collected in 2008, on the lowest line of each microplate.

Bradford protein [18] was determined for untreated control samples applying 50 µl Bio-Rad Protein Assay Reagent dye, binding to protein amino acid residues, in 200 µl sample extract diluted in PBS (1∶1). The colour reaction was read with a microplate reader at 590 nm after 5 min. A standard curve was produced with 0–5 µg BSA protein.

### Statistics

Data were analyzed with the statistical software JMP 7 (SAS, Cary, NC, USA). Linear contrasts were used to test for overall effects of any of the treatment levels compared to the control. A regression analysis was used to test for the shape of the relationship between glomalin production and increasing stress levels.

## Results

**Figure 1:**
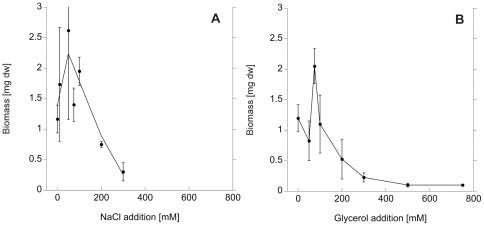
AM fungal mycelium biomass under salt- and osmotic stress. Mycelium biomass [mg dw] of *G. intraradices* in the fungal compartment in *in vitro* culture, as a response to rising salinity induced by NaCl addition (a) and osmolarity as induced by glycerol addition (b) into the growth medium.

**Figure 2:**
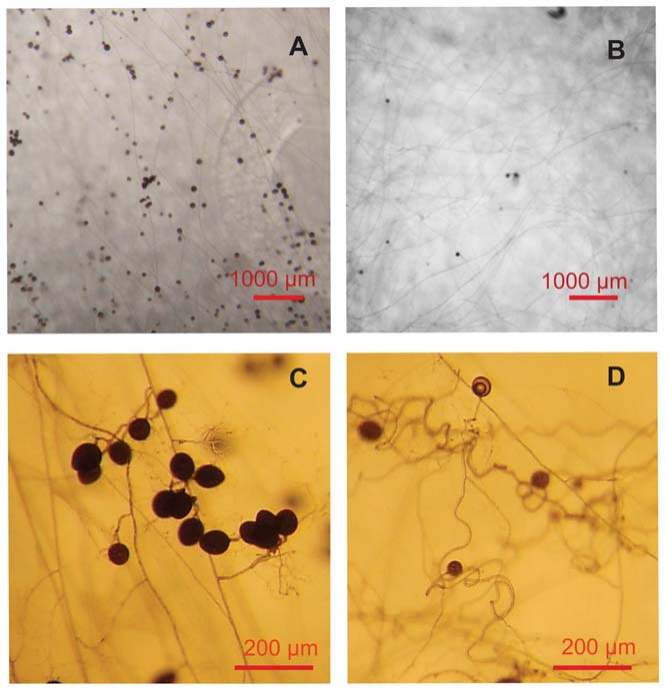
Growth morphology of *G. intraradices* under high osmolarity. Growth morphology of *G. intraradices* growing in untreated M-medium in *in vitro* culture (a, c) and under high osmolarity (b, d), as here under 250 mM glycerol addition. Scale bars 1000 µm (a, b) and 200 µm (c, d). Spore abundance and diameter are reduced, and hyphae show curly growth morphology under high glycerol addition.

Mycelium biomass was affected both by addition of NaCl and glycerol (Fig. 1A, 1B). Biomass initially increased but declined above a threshold level of around 100 mM. In the NaCl treatment, no hyphal growth was recorded at concentrations higher than 300 mM, which corresponds to an osmolarity of 600 mOsm. In the glycerol treatment, some hyphal growth was still found at 750 mM (corresponding to 750 mOsm), but only in one out of four replicates. High levels of glycerol influenced spore diameter and abundance, being less than half the size or absent at 300 mM glycerol or higher (Fig. 2A, 2B). High osmolarity in the medium caused altered growth morphology, hyphae were growing in a curly way instead of the typical straight runner hyphae and absorbing structures (Fig. 2C, 2D). The biomass of the mycelium exposed to the clipping treatment was not significantly different from the control, since the clipping was performed after development of the mycelium.

**Figure 3:**
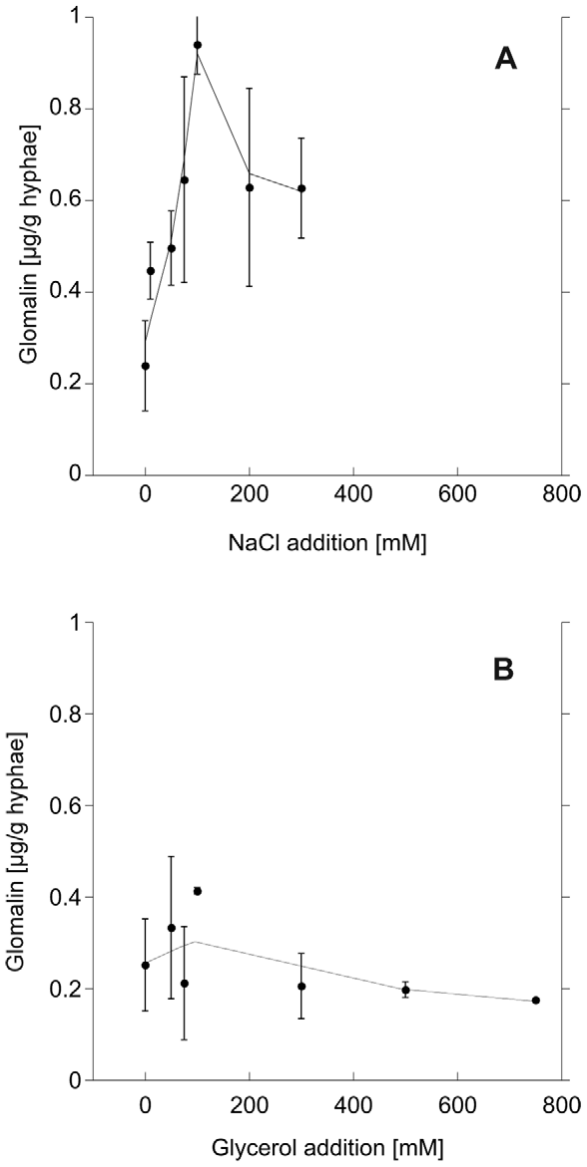
Glomalin production under salt- and osmotic stress. Glomalin production per mg hyphal dry weight in *G. intraradices* in *in vitro* culture, as a response to rising salinity as induced by NaCl addition (a) and rising osmolarity as induced by glycerol addition (b) into the growth medium.

**Figure 4:**
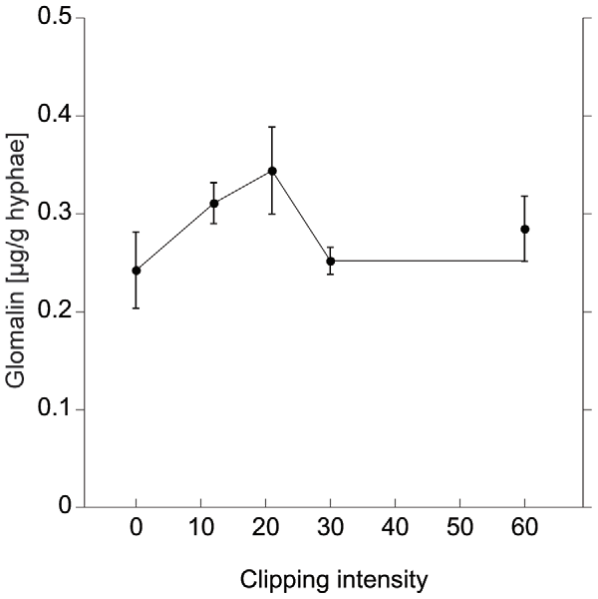
Glomalin production after grazing simulation. Glomalin production per mg hyphal dry weight in *G. intraradices* in *in vitro* culture, as a response to rising intensity in mechanical injury of the hyphae, induced by clipping. The injuries had to be undertaken on an existing mycelium and was therefore not present since experimental start as in the salinity and osmolarity treatments. Intensities should therefore not be directly compared.

The effect sizes in the various treatments on glomalin production differed. NaCl addition led to a strong, up to 5-fold increase (F = 24.6, p<0.0001; Fig. 3A); while glycerol addition, simulating water stress, did not led to increased glomalin production (F = 0.07, p = 0.79; Fig. 3B). The clipping treatment led to a slight, marginally significant increase of glomalin in the hyphae (F = 3.95, p = 0.057; Fig. 4), which was elevated at its peak (20 clippings) 30% compared to the control.

The increased glomalin production under rising NaCl addition followed a second order polynomial function (R^2^ = 0.41, p = 0.02, Fig. 3A). Also the clipping treatment showed a trend towards elevation at intermediate treatment levels (Fig. 4).

Spore to hyphal ratio was not influenced by the NaCl or clipping treatment (p = 0.93 and p = 0.42, resp.). Glomalin levels were not sensitive to the ratio of spores to hyphal biomass or to spore abundance in the NaCl- and clipping treatment series (p = 0.64, and p = 0.18, resp.). However, with increasing glycerol addition we observed decreased spore number and diameter (R^2^ = 0.56, p>0.0001) in parallel to the decreasing glomalin levels (p = 0.08).

In control plates not subjected to any treatment, autoclave-stable Bradford protein content was 10–15 µg mg^−1^ hyphae, while immonureactive protein was 0.1–0.2 µg mg^−1^ hyphae, i.e. 1–2% of the total autoclave-stable protein. The amount of glomalin was not influenced by the root biomass in the root compartment, and root biomass was not influenced by the different treatments.

## Discussion

We found a strongly increased glomalin production in hyphae of *G. intraradices* under NaCl stress, and thus partly confirmed our main hypothesis. But even though salinity stress includes water stress, the response of glomalin production to salt and low water potential was substantially different. This suggests that a certain physiochemical property of NaCl might induce the response, e.g. direct toxicity of the ions. Already the lowest level of NaCl addition (10 mM) caused a strong response in glomalin production. The Na concentration of the M medium is 0.5 µg/ml, which corresponds to 0.022 mM. The addition of 10 mM (230 µg/ml) is therefore a 460-fold increase in Na concentration. The 10 mM addition does not represent a severe change in overall osmolarity since the base levels of ionic strength in the liquid M medium is already around 13 mOsm. Under high salinity stress, cells need to discriminate against Na^+^ ions, which are toxic for cellular reactions. Na ions are taken up simultaneously with K due to its physiochemical similarity, and can interfere with enzymatic reactions and with charge balancing of macromolecules like proteins and nucleic acid [19]. *G. intraradices* has previously been shown to be moderately salt tolerant [20], and AMF have been found to have ability to selectively take up nutrients and discriminate against toxic Na ions [21]. Heat shock proteins are typically multifunctional; their expression is upregulated under biological, chemical and physical stress, and an important feature is their chaperone activity, i.e. the prevention of misfoldings of proteins [22]. Glomalin as a putative heat shock protein could be involved in minimizing possible cytosolic damages of Na induced protein misfolding [19], which other ions like K do not induce.

Soil aggregation, an ecosystem function with which glomalin-related soil protein concentrations are correlated, is highly disturbed under high NaCl levels. Na ions in the soil solution cause the soil colloids to repel from each other, leading to flocculation, the inability of the colloids to aggregate. A higher level of glomalin production could counteract this phenomenon even if it is not actively secreted into the soil but reaching it after hyphal death. [23] found a negative relationship of the amount of GRSP in mangrove estuary soils and (among other factors) their salinity. This is, however, not necessarily a contradiction to our results. The positive relationship between salinity and glomalin found by us was per mg mycelium. The total amount of glomalin per plate harvest was declining under highest levels of NaCl, since the biomass of *G. intraradices* was declining with rising salinity, as previously found for G intraradices in sterile culture [20], and as AMF abundance declines in general in saline soils [24].

It was unexpected that the simulation of osmotic stress did not result in a similar reaction in glomalin production as the salinity stress did, since salinity stress includes osmotic stress. Glycerol was chosen as an osmoticum since it can be found in numerous fungi as a compatible solute synthesized under water stress conditions (e.g. in ectomycorrhizal fungi, [25]), can be isolated from AM fungi [26], and is generally considered to be non-toxic and neutral to other cell metabolic pathways. High osmotic stress that is not caused by mineral ions forces organisms to produce low-molecular weight carbohydrates as internal osmoregulators. Glycerol itself is a typical osmoregulator, but AMF likely cannot take up the molecules via their extraradical hyphae since AMF are obligate biotrophs [27]. Osmoregulation under high external molecule concentration such as in our glycerol treatments is therefore costly [28], and the low biomass, low spore abundance and diameter caused by glycerol addition might indicate strong C limitation in the fungus. [29] found a consistent positive relationship between the amount of available carbon to AMF and GRSP levels in different ecosystems, and thus the need to produce osmoregulators might compete with glomalin production for C resources. Our results suggest that glomalin is not involved in amelioration of osmotic stress. Instead, [30] found that the gene for another heat shock protein, a luminal binding protein in the endoplasmatic reticulum, was upregulated in *G. intraradices* under drought stress.

We found a marginally significant reaction of glomalin production in *G. intraradices* to the clipping treatment. The stress was not continuously present from the start of the experiment like the drought and salinity stress, but was applied on already established parts of mycelium. Therefore, effect sizes of glomalin response should not be directly compared to the ones in the NaCl and glycerol treatment. The artificial injuries simulated grazing in the sense that it potentially physically disrupted the flux of nutrients in the mycelium front. Note that our treatment only involved the mechanical damage but no possible chemical communication by collembolans or other grazers, and therefore it would be interesting to also examine the effect of real grazing soil biota. The disruption of the extraradical mycelium can be detrimental to AMF since C and mineral nutrients are taken up at opposite ends of the mycelium (i.e. the host plant cells and the hyphal tips, respectively) and need to be transported over long distances to the sink at the other end. Collembolan grazing has been demonstrated to disrupt the carbon flow in AMF networks significantly [31], and we expect a high selection pressure for grazing avoidance, since the lack of C to maintain and extend the mycelium front will impede symbiotic function. Other soil fungi are known to possess repellent metabolites or crystalline structures at the hyphal surface [32], but no defence strategy for AMF is known so far. In addition to numerous studies that showed that AMF were not a preferred food source, [33] and [9] showed that collembolan fecundity was reduced when AMF were the only food source. We still lack a mechanistic understanding of these patterns. In our study we found only a slight increase in glomalin production when mycelium was exposed to simulated grazing: this can be interpreted as a hint that this protein may be involved in mediating grazing interactions, or at least in grazing related stress responses, but this does not constitute conclusive evidence. The substance may also be constitutively expressed and only minimally upregulated when the mycelium is actually exposed to damage by grazers.

We conclude that glomalin production is triggered by salinity stress and possibly by grazing stress, but not all general kinds of stresses induce a higher glomalin production.
